# Seismic constraints on glacier density

**DOI:** 10.1038/s41598-025-26440-z

**Published:** 2025-11-26

**Authors:** Ariane Lanteri, Scott Keating, Lars Gebraad, Sara Klaasen, Marta Pienkowska-Côte, Olaf Eisen, Andrea Zunino, Kristín Jónsdóttir, Coen Hofstede, Dimitri Zigone, Andreas Fichtner

**Affiliations:** 1 Institute of Geophysics, ETH Zurich, Zurich, Switzerland; 2 Alfred Wegener Institute, Helmholtz Centre for Polar and Marine Research, Bremerhaven, Germany; 3 Faculty of Geosciences, University of Bremen, Bremen, Germany; 4 Institut Terre et Environnement de Strasbourg, Université de Strasbourg, Strasbourg, France; 5 Icelandic Meteorological Office, Reykjavik, Iceland

**Keywords:** Climate sciences, Planetary science, Solid Earth sciences

## Abstract

Terrestrial ice bodies are important regulators of climate and sea level variations. They influence the water cycle, provide fresh water and energy for human society, and contribute to the living basis of numerous ecosystems. Understanding the structure and dynamics of land ice requires knowledge of its mass density, which is essential for ice core climatology and estimates of mass balance components, such as mass loss, ice discharge and surface melt. We combine densely sampled fiber-optic sensing data from strong serendipitous anthropogenic sources with Hamiltonian Monte Carlo sampling to extract direct seismic constraints on firn density (i.e. the transitional layer between fresh snow and glacial ice). Our approach avoids biases introduced by subjective regularization choices, does not require empirical scaling relations from seismic wave speeds to density, and provides reliable uncertainty estimates. We demonstrate that high-quality surface-wave overtone data can directly constrain density to around 100 m depth. Commonly used scaling relations from seismic wave speeds to density, however, fail to reproduce resolvable details of glacial density structure, and they tend to deviate from direct constraints on the order of ±10 %. Consequently, ice mass inferred from seismic wave speed may be incorrect by a similar amount.

## Introduction

Glaciers in a broad sense, including ice sheets and ice caps, are critical elements of the Earth’s cryosphere. They influence the water cycle, sea level and climate, provide the living basis for numerous ecosystems, and serve as fresh water and energy reservoirs for human society. Glaciers are dynamic systems, controlled by a complex interplay of snow accumulation, melting, refreezing, compaction and various other processes. Understanding the structure and dynamics of glaciers requires detailed knowledge of their mass density distribution, which enters calculations of ice mass balance, surface melt, ice discharge and the resulting contribution to sea level change^1–4^. Furthermore, estimates of glacier density are essential to constrain the temporal resolution of climate information in ice cores^5^.

The direct measurement of density in snow pits or boreholes is time-consuming and laborious, and it only provides point-localized constraints. Seismic methods may be considered more efficient alternatives that probe a larger volume, although they come with their own set of limitations. Body wave travel times only constrain seismic wave speeds, and the sensitivity of surface wave dispersion to density is often considered insufficient. Limited seismic data coverage and quality, combined with the impact of subjective regularization choices typically prevent the reliable extraction of density information. Although the emergence of full-waveform inversion methods spawned new hopes that density may be constrained by seismic data^6,7^, substantial trade-offs with other parameters still pollute density models obtained by gradient-based optimization methods^8^. A multi-parameter variant of the Backus-Gilbert method recently suggested that higher-mode surface wave data contain information on density^9^, but requires the linearization of an inverse problem that is known to be nonlinear. To partly circumvent these issues, scaling relations based on more robust seismic wave speed models are often used to infer density^10,11^. However, having been developed for data from specific sites, they do not account for regional variations that may be expected in response to different climatic conditions, including air temperature and snow accumulation throughout the year. Moreover, wave speed to density scaling relations do not account for seismic anisotropy, thereby introducing an ambiguity as to which wave speed is supposed to be converted to density. A recent comparison of seismic velocity and firn core density profiles at the South Pole indicates that widely used scaling relations may underestimate firn air content by as much as 15%^12^.

In the light of recent developments in seismic instrumentation and inversion methods, we revisit the question whether vertical density variations may be constrained directly by seismic data, without the need for scaling relations.

Distributed Acoustic Sensing (DAS) converts fiber-optic cables into an array of seismic sensors with meter-scale spacing^13,14^. The recording bandwidth from mHz to kHz^15,16^, combined with the relative ease of deployment at the surface or in boreholes, makes DAS an attractive alternative to seismometers in cryosphere research^17^. Covering the cable with snow or trenching it a few tens of centimeters into the ice, often provides sufficient coupling and wind shielding to produce data that compete in quality with geophone recordings, though with much denser spatial sampling. Using either active sources or seismic noise, DAS has been used to constrain the structure of firn and the underlying ice, including attenuation and anisotropy^12,18–23^. Furthermore, DAS has enabled studies of glacial seismicity, including basal stick-slip events^24,25^, earthquake-induced ice shelf resonance^26^ and englacial ice quake cascades^27^.

Originally developed for quantum chromodynamics simulations^28^, Hamiltonian Monte Carlo (HMC) has been discovered as an efficient alternative to Metropolis–Hastings-type algorithms for the solution of Bayesian inference problems^29,30^. By exploiting derivative information, HMC is able to explore high-dimensional model spaces, which makes it particularly attractive for geophysical inverse problems^31–35^. Unlike inverse problem approaches based on some form of least-squares inversion or iterative optimization, HMC does not require any regularization to avoid instabilities or the appearance of artifacts. Honest prior information can therefore be translated into reliable uncertainty estimates that are not biased by subjective regularization choices. Furthermore, the full nonlinearity of the inverse problem can be taken into account.

Here we explore the combined use of DAS and HMC to extract direct constraints of seismic data on glacier density. For this we consider two independent datasets collected on the Greenland Ice Sheet and the Vatnajökull Ice Cap (Iceland). Both have been produced serendipitously by anthropogenic activity, that is, without special planning or the need of active seismic sources. The datasets represent different glacial environments, thereby highlighting the range of applicability of our method.

## Datasets

**Fig. 1:**
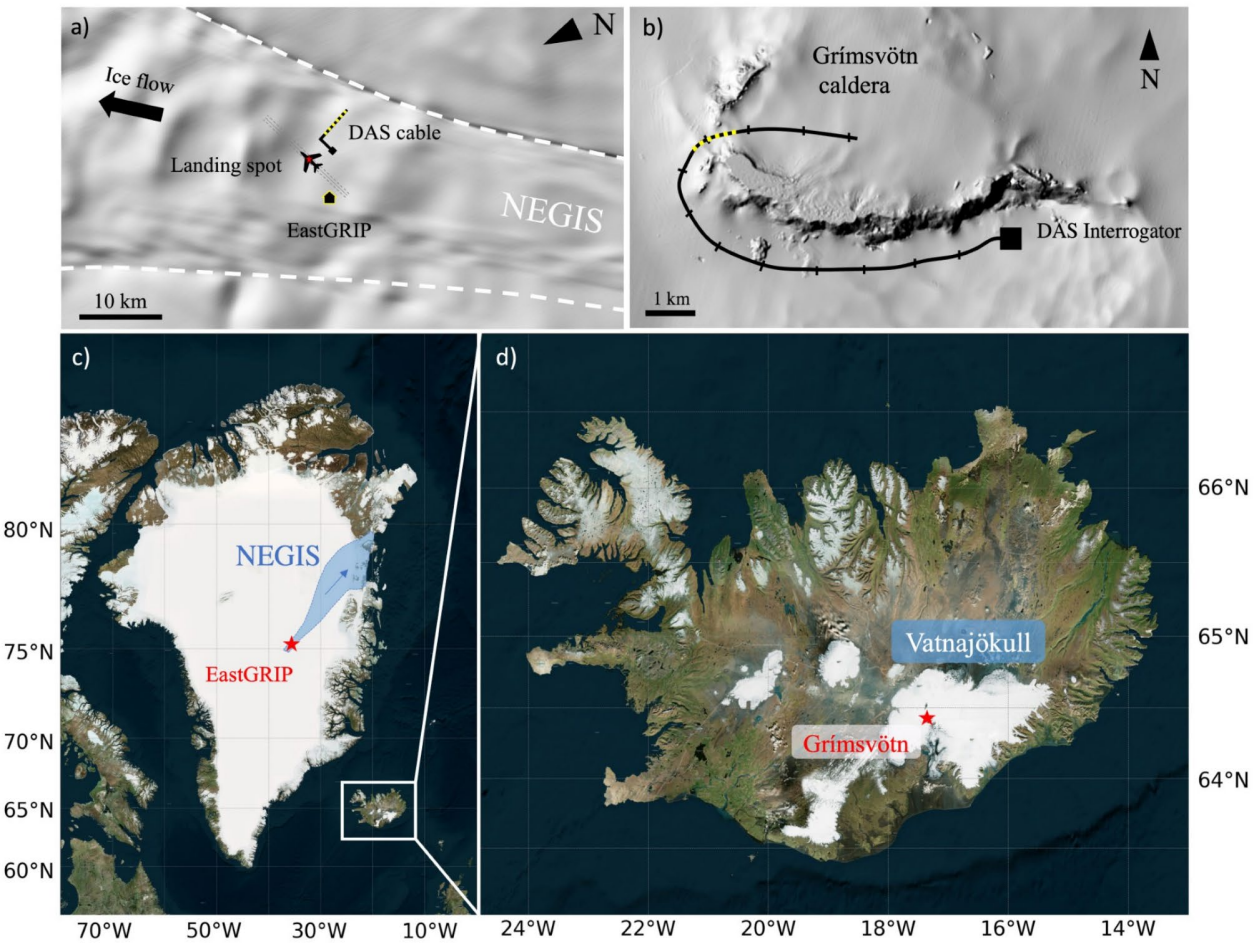
Setups of the DAS experiments on NEGIS (EastGRIP site, Greenland) and Vatnajökull (Grímsvötn, Iceland) that produced the data analysed in this study. The cable segments for which we analyzed data are highlighted by the yellow-black dashed lines on the topographic maps^44^ in panels (**a**) and (**b**). Background elevation data are from the ArcticDEM 2024 mosaic, courtesy of the Polar Geospatial Center, University of Minnesota. The regional-scale geographic location is indicated by the red stars in panels (**c**) and (**d**) (OpenStreetMap contributors 2015).

**Fig. 2:**
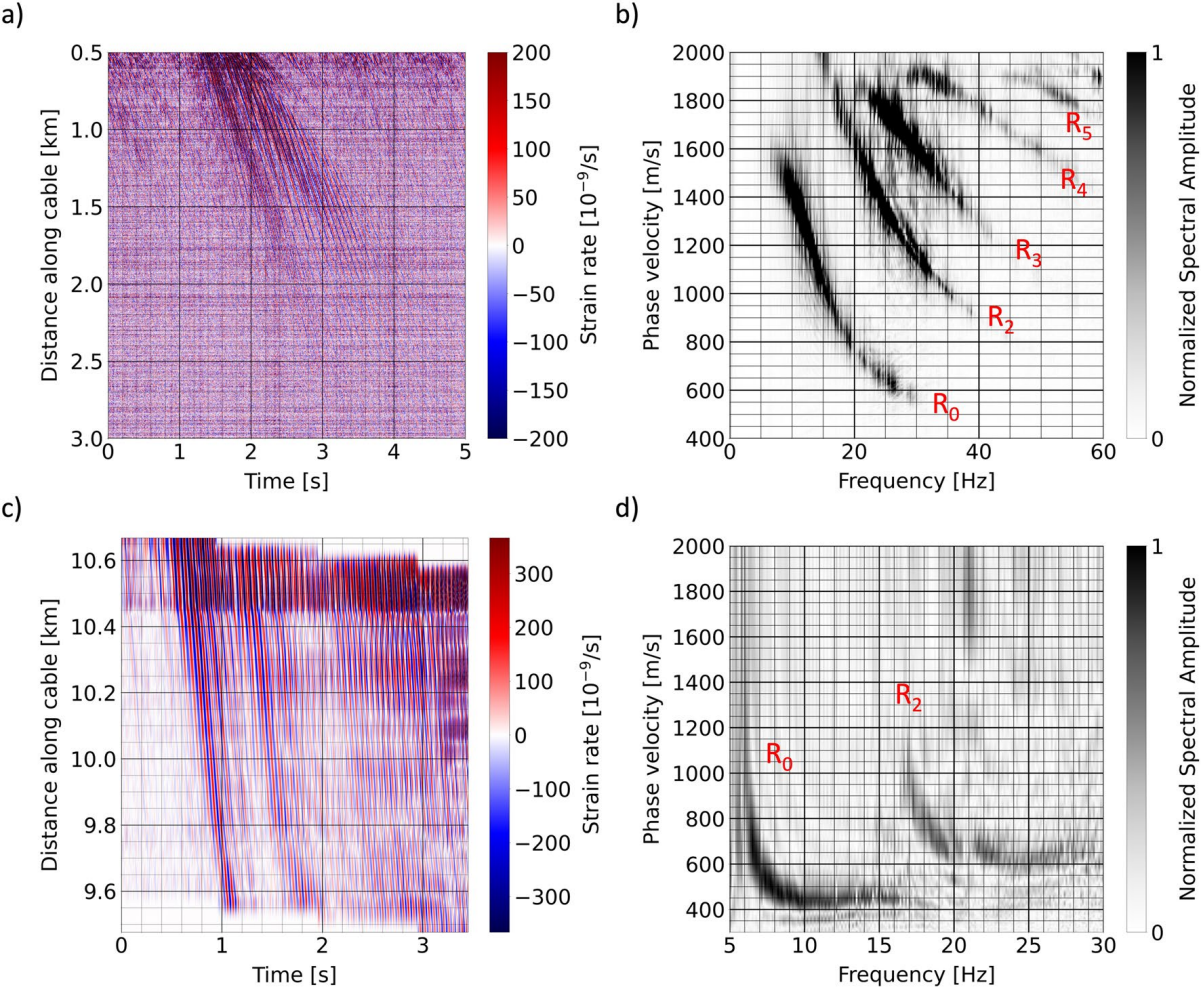
Summary of DAS recordings used in this study. (**a**) Space-time domain recording of the seismic waves excited by an airplane landing near the EastGRIP site, starting at UTC 17:02:08 on 26 July 2022. (**b**) Frequency-phase velocity representation of (**a**) obtained by a 2D Fourier transform. Rayleigh modes identified with the help of surface wave dispersion modeling are labeled in red. They include the fundamental mode, $\hbox {R}_0$, and the second to fifth overtones, $\hbox {R}_2$ to $\hbox {R}_5$^21^. (**c**) Space-time domain recording of seismic waves excited by a snow groomer on Grímsvötn, starting at UTC 19:33:07 on 13 April 2021. The diagonal step pattern is the result of time windowing, which eliminates the static displacement of the snow groomer and emphasizes the seismic wave part of the data. (**d**) Frequency-phase velocity representation of (**c**) computed by MASW. The two easily visible modes are the fundamental mode, $\hbox {R}_0$, and the second overtone, $\hbox {R}_2$.

The Greenland DAS data were collected with a fiber-optic cable of 3 km length, of which $\sim$2.5 km were linear and perpendicular to the landing strip of the East Greenland Ice Core Project (EastGRIP) on the Northeast Greenland Ice Stream (NEGIS), as illustrated in Fig. 1a,c. The touch down of a transport airplane served as a powerful seismic source that produced recordings with a signal-to-noise ratio well above 10 in the frequency range from 5–60 Hz^21^. A 2D Fourier transform of the space-time domain data shown in Fig. 2a, produces the phase velocity-frequency representation in Fig. 2b, which contains multiple, clearly visible Rayleigh wave modes.

Reflectivity modeling revealed that the first overtone, $\hbox {R}_1$, is only weakly excited. Mistaking the second overtone $\hbox {R}_2$ for $\hbox {R}_1$ would produce entirely implausible wave speed profiles, including rapidly oscillating wave speed profiles and pronounced low-wave speed layers at depth^21,36^. Hence, the fundamental mode, $\hbox {R}_0$, and the second to fifth overtones, $\hbox {R}_2$ to $\hbox {R}_5$, dominate the wavefield. Around the EastGRIP site, the ice thickness is around 2700 m, and the medium can be considered laterally homogeneous at the scale of the experiment^37,38^.

In Iceland, we deployed a 12.5 km long fiber-optic cable around and inside the subglacial volcano Grímsvötn, covered by Europe’s largest ice cap, Vatnajökull^39,40^. Shown in Fig. 1b,d, the experiment was primarily intended to record glacial seismicity and volcanic tremor. However, the DAS system also captured the wavefield excited by snow groomers (PistenBully snow trucks), used to trench the cable and transport equipment. Typical snow groomer DAS data in the space-time domain are displayed in Fig. 2c. Multi-channel analysis of surface waves (MASW)^41,42^ produces the frequency-phase velocity representation of Fig. 2d, which reveals the presence of two Rayleigh wave modes between 7 and 28 Hz. Similar to the EastGRIP data, forward modeling for a wide range of plausible wave speed profiles helps to identify the modes and reveals that the first Rayleigh overtone is again only weakly excited. The mode branch above $\sim$17 Hz is the second Rayleigh overtone $\hbox {R}_2$, and mistaking it for $\hbox {R}_1$ produces exotic wave speed variations with depth. Radio-echo sounding produced estimates of ice layer thickness around 100–150 m below the cable segment of interest (9.4–10.6 km along the cable)^43^.

Both datasets, and the one from EastGRIP in particular, are characterized by exceptional data quality that has mainly three contributions: (i) The cables were trenched 0.5–1 m into the ice, using a snow groomer near EastGRIP^21^ and a purpose-built trenching sled on Grímsvötn^39^. This provided shielding from wind noise and nearly perfect coupling to the surrounding medium. (ii) Compared to commonly used explosive sources, the airplane touch down and the $\sim 5\, 000$ kg snow groomer were more powerful and long-lasting sources that produced high-amplitude surface wave trains. (iii) The dense and regular spacing of hundreds to thousands of DAS channels helps to suppress incoherent noise in the calculation of the frequency-phase velocity representations.

The two experimental sites on NEGIS and Vatnajökull, respectively, are characterized by differences in the ice structure that originate from their respective climatic regimes. Around the EastGRIP site, snowfall throughout the year and temperatures that are mostly below $0^\circ$C contribute to a positive mass balance and the formation of a 60–70 m thick firn layer^21,37^. In contrast, Vatnajökull experiences a warmer climate with limited snow accumulation in winter and significant melting during the summer months, which leads to sharper stratification compared to the EastGRIP site. These structural differences constitute prior knowledge that influences the model parametrization and the choice of an initial model, as explained in more detail below.

## Methods

The dispersion of surface wave modes, shown in Fig. 2b and d, carries information on subsurface structure, which can be estimated by inverting the vector of observed data, $\textbf{d}^\text {obs}$, for a vector of model parameters, $\textbf{m}$. The latter contains the elastic properties of a stratified radially anisotropic medium, namely the speeds of the vertically and horizontally polarized S waves ($v_\text {SV}$, $v_\text {SH}$), the speeds of the horizontally and vertically propagating P waves ($v_\text {PH}$, $v_\text {PV}$), the anisotropy parameter $\eta$ that controls the dependence of P wave speeds on the incidence angle, and density $\rho$ in the different layers^45,46^. To quantify the fit between the observed dispersion data $\textbf{d}^\text {obs}$ to those predicted by $\textbf{m}$, we need to solve the nonlinear forward problem $\textbf{d} = \textbf{g} (\textbf{m})$ of computing synthetic data $\textbf{d}$.

### Bayesian inference and Hamiltonian Monte Carlo

Constraining parameters such as $\rho$, that are critical for glaciological studies but only have a smaller influence on Rayleigh wave dispersion data than $v_\text {SV}$ and $v_\text {PV}$, requires the use of an inversion method that minimizes biases introduced by subjective regularization, provides reliable uncertainty estimates and accounts for nonlinearity of the forward operator $\textbf{g}$. These objectives can be achieved with the help of Bayesian inference implemented by Monte Carlo methods^47,48^. In Bayesian inference, all information is encoded in probability densities, including prior knowledge on model parameters, $p(\textbf{m})$, and prior information on the observed data, $p(\textbf{d}^\text {obs}|\textbf{m})$, which measures the discrepancy between $\textbf{d}^\text {obs}$ and the computed data $\textbf{d}=\textbf{g}(\textbf{m})$ for a selected model $\textbf{m}$. Bayes’ theorem combines the prior information to provide the posterior information on model parameters in the form of the probability density

1 $$\begin{aligned} p(\textbf{m}|\textbf{d}^\text {obs}) = k\, p(\textbf{d}^\text {obs}|\textbf{m})\,p(\textbf{m})\,. \end{aligned}$$

The constant *k*, called the evidence, ensures that $p(\textbf{m}|\textbf{d}^\text {obs})$ integrates to 1. In the absence of closed-form solutions of the inverse problem, approximations of the posterior distribution can be obtained with Markov chain Monte Carlo (McMC) methods^47,48^. A critical aspect of all McMC methods is the number of samples required to obtain useful estimates of $p(\textbf{m}|\textbf{d}^\text {obs})$. For algorithms of the Metropolis–Hastings family, the number of required samples scales as $N^2$, where *N* is the dimension of the model space^49^. This poor scaling often prevents the solution of higher-dimensional problems. HMC improves this scaling to $N^{5/4}$, which enables the solution of geophysical inverse problems that cannot be addressed with Metropolis–Hastings^29^. The better scaling of HMC results from the exploitation of derivative information, which allows the algorithm to approach relevant models more quickly and to produce a chain of samples that are more independent. For more technical details on HMC, readers are referred to the extensive body of applied mathematics and geophysical literature^30–33,50,51^. In this work, we implement the forward problem of computing surface wave dispersion curves using the well-established formulation of Takeuchi and Saito^45^ and use the HMCLab code package^35^ for sampling.

### Model parametrization and prior information

**Fig. 3:**
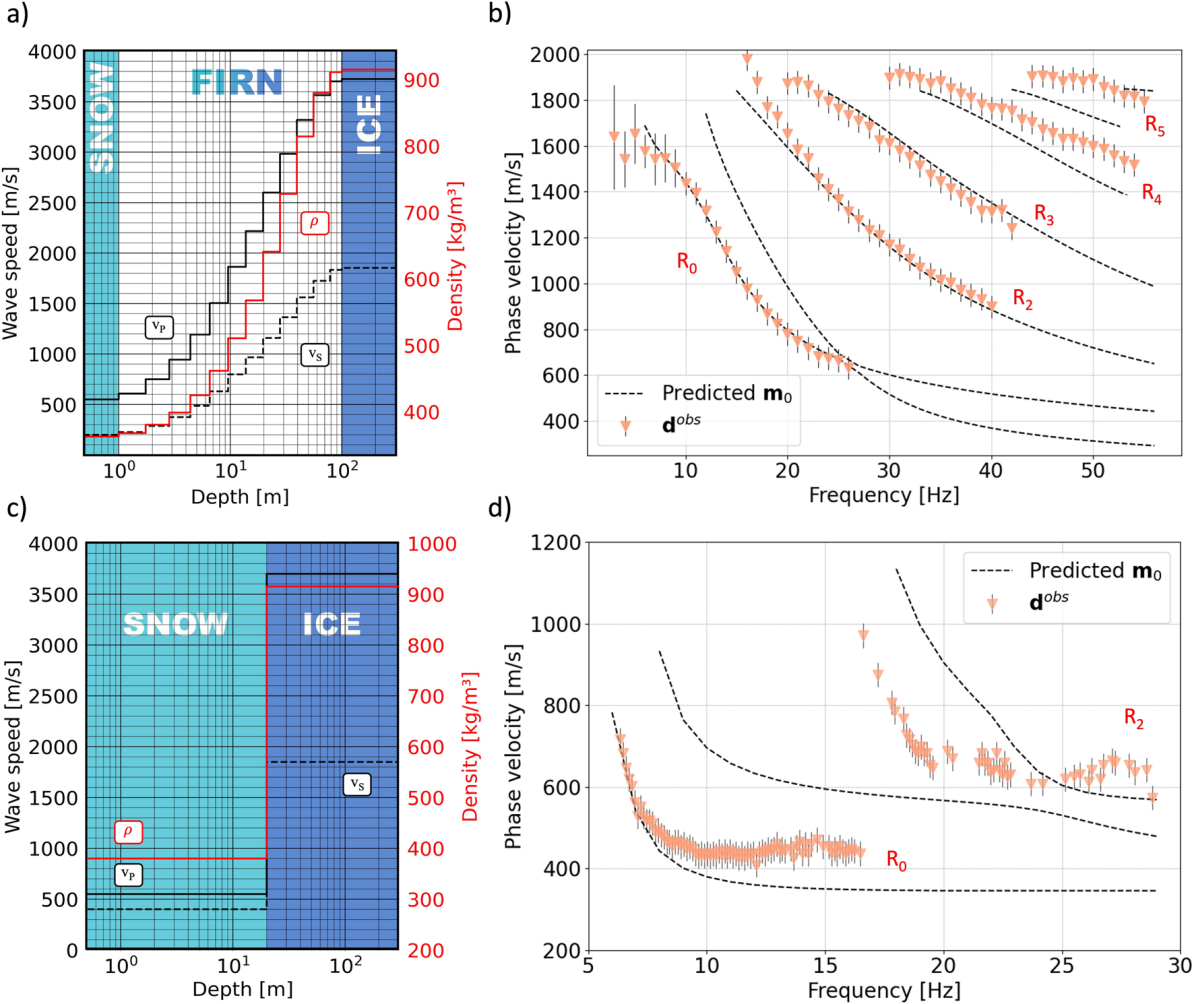
Isotropic prior maximum-likelihood models and initial data fit. (**a**) The prior maximum-likelihood model for the EastGRIP data is composed of 14 layers of increasing thickness. Each layer is characterized by its density $\rho$ (red, right axes) and the isotropic P- and S-wave speeds ($v_\text {P}=v_\text {PH}=v_\text {PV}$, $v_\text {S}=v_\text {SH}=v_\text {SV}$, black, left axes). The model largely explains the fundamental-mode and second-overtone data in (**b**). Dashed lines are computed dispersion curves, and orange triangles with black solid lines represent phase velocity measurements and their error standard deviations estimated from the half width of the frequency-phase velocity representations in Fig. 2b,d. Panels (**c**) and (**d**) show the prior maximum-likelihood model and data fit for the Grímsvötn data. Overlying a single ice layer below 20 m depth, the top three layers (0–2 m, 2–8 m, 8–20 m depth) have identical parameter values, and therefore appear as a single layer in the prior model.

The posterior mean of a previous deterministic inversion of the airplane-landing data at EastGRIP^21^ serves as a plausible prior maximum-likelihood model for $v_\text {SV}$. By starting with the prior assumption of an isotropic medium, and using common scaling relations for wave speeds and density^10,11^, we can construct complete set of prior maximum-likelihood parameters, shown in Fig. 3a. We discretize the model space in 14 layers with linearly increasing thickness to a maximum depth of 120 m. By trial and error, we found that this is a minimum number of layers that allows the prior model to fit the fundamental-mode data to within the observational errors, and the linear increase of layer thickness is motivated by the previously inferred linear increase in resolution length^21^. Below 120 m depth, the sensitivity of the surface wave with the lowest usable frequency of $\sim$5 Hz has vanished, meaning that layers at greater depth would merely waste computational resources. As illustrated in Fig. 3b, this initial model explains most of the fundamental-mode and second-overtone data to within their observational errors, estimated as the half-width of the dispersion images in Fig. 2. With six model parameters for each of the 14 layers, the model space dimension is 84. To complete the construction of the model space prior $p(\textbf{m})$, we assume a Gaussian distribution, with mean equal to the maximum-likelihood parameters shown in Fig. 3a and a standard deviation equal to 10 % of the mean at a given depth. These relatively tight bounds agree with the posterior uncertainties inferred from the earlier Backus-Gilbert inversion of the same dataset^21^ and largely results from the large volume of dispersion measurements with small errors in the range of few tens of m/s.

For the Vatnajökull ice cap, our prior knowledge on subsurface structure from previous studies at the experiment site is sparse. A core drilled at several kilometers distance on the Grímsvötn ice shelf in 1993^52^ located the solid ice interface around 22 m depth. To obtain a useful model parametrization, we conducted an extensive manual trial-and-error study where we varied the number of layers and the depth firn-ice interface. We determined that at least three layers for the firn, overlying a fourth layer below 20 m depth that represents solid ice, are needed to fit the data to within the observational errors. In this process, we also constructed the prior maximum-likelihood model in Fig. 3c, where the top three layers have identical parameter values. This model explains the fundamental-mode data to frequencies of $\sim$10 Hz to within their errors, as shown in Fig. 3d. Having six free parameters in four layers, the model space dimension is 24. To reflect our poor prior knowledge in the design of the model space prior $p(\textbf{m})$, we again assume a Gaussian distribution but assign a larger standard deviation of 20 % of the parameter values at a given depth.

## Results

We applied HMC sampling to the dispersion curve data from EastGRIP and Grímsvötn, using $500\,000$ samples for both scenarios. We designed the mass matrix based on the prior model covariance to ensure rapid convergence of the HMC sampler. As the chains approach convergence, the distribution of samples increasingly approximates the true posterior and becomes effectively independent of the initial mass matrix choice. Although convergence can never be strictly guaranteed, we mitigated this limitation by using more than twice the number of samples required to obtain stable and reproducible results. The results in the broadest sense consist of all posterior samples, from which all available information on seismic material properties can be extracted. Here we limit ourselves to the presentation of a subset of the results, focusing on three specific aspects: the importance of higher-mode data, the possibility to constrain density variations with depth, and the validity of previously derived scaling relations from seismic wave speeds to density. In our presentation, we omit two of the six parameters, $v_\text {SH}$ and $\eta$. Sensitivity of the former is zero, and information on the latter is so weak that the posterior does not differ from the prior distribution. The ensemble of all samples is available as supplementary material.

### Higher-mode constraints on density

**Fig. 4:**
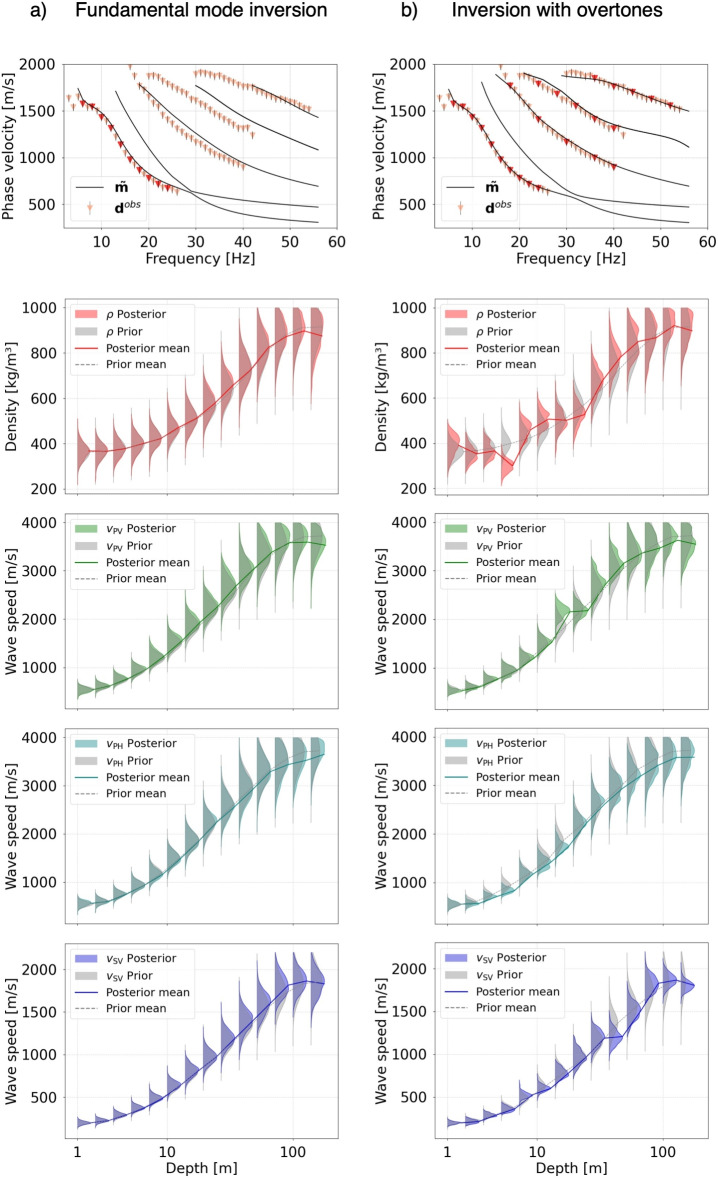
Inversion results for the EastGRIP dataset. In the top panels, red triangles with vertical black lines indicate the phase velocity observations that have been used in the inversion. Solid black curves are the dispersion curves computed with the posterior mean model $\mathbf {\tilde{m}}$. (**a**) Posterior data fit and 1D marginals when only fundamental-mode observations are used. The distributions closely follow the approximately exponential prior. (**b**) The same as in (**a**) when overtone data are included.

Figure 4a summarizes the inversion results for the EastGRIP data when only the fundamental-mode observations are used. The posterior 1D marginal distributions of $\rho$, $v_\text {pv}$, $v_\text {ph}$, $v_\text {sv}$, $v_\text {sh}$ closely follow the prior distributions. They are approximately Gaussian and have similar posterior means standard deviations as the prior. This result confirms the expectation that the fundamental-mode data alone do not add significant information to the prior model obtained from the deterministic inversion of the airplane landing data^21^. Although the posterior mean model, $\tilde{\textbf{m}}$, explains the fundamental-mode data mostly to within their errors, it does not succeed in matching the overtone observations.

Adding the overtone data produces a posterior mean model that correctly explains their dispersion characteristics and also leads to substantial modifications of the posterior distributions, as illustrated in Fig. 4b. The incorporation of overtones provides additional sensitivity, notably to density—which is otherwise difficult to constrain—and helps reduce non-uniqueness in the inversion. It results in posterior distributions that are significantly different from the prior, obtained by scaling the wave speeds from the earlier deterministic inversion of the multi-mode data^21^. For many of the depth intervals, the posterior distributions become significantly non-Gaussian, which attests to the importance of nonlinearity in this inverse problem. It also adds significant deviations from the approximately exponential density and wave speed profiles. In particular, the density profile contains a shallow high-density layer between the surface and 1 m depth that plausibly results from compaction by the snow groomer during the process of cable trenching. As a consequence, the 1–7 m depth interval appears as a low-density layer.

**Fig. 5:**
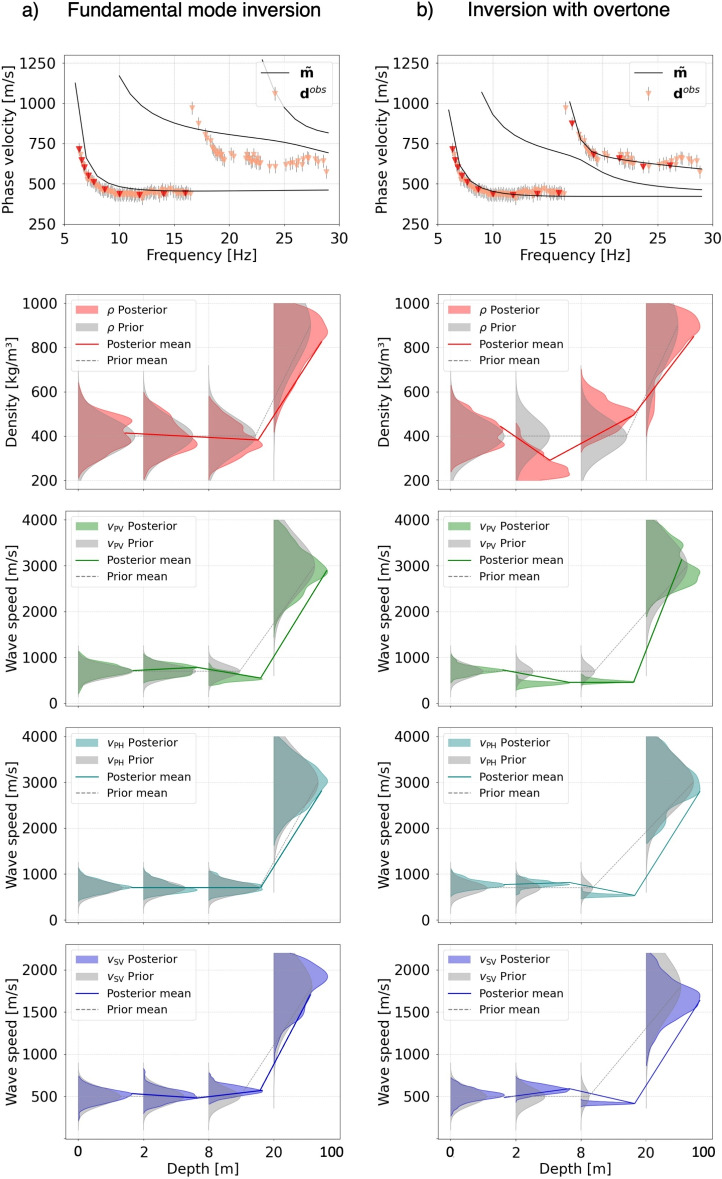
Inversion results for the Grímsvötn dataset. In the top panels, red triangles with vertical black lines indicate the phase velocity observations that have been used in the inversion. Solid black curves are the dispersion curves computed with the posterior mean model $\mathbf {\tilde{m}}$. (**a**) Posterior data fit and 1D marginals when only fundamental-mode observations are used. The distributions slightly deviate from the prior within the firn layer. (**b**) The same as in (**a**) when overtone data are included.

Similar results for the Grímsvötn data are presented in Fig. 5. In the absence of overtone observations, the posterior distributions for all parameters are broad and similar to the prior distributions, indicating that fundamental-mode data do not add substantial information to our trial-and-error guess. When overtone observations are included, all posterior distributions become more peaked. This effect is most pronounced for the wave speeds within the firn layer. This result can be explained by efficient wave trapping within the firn layer, characterized by waves speeds that are a factor of $\sim$3 lower than in the underlying ice. As for the EastGRIP data, many of the posterior distributions become significantly non-Gaussian, and a low-density layer around 2–10 m depth appears in response to near-surface compaction caused by the snow groomer driving over the cable after trenching.

### Scaling relations from seismic wave speeds to density

**Fig. 6:**
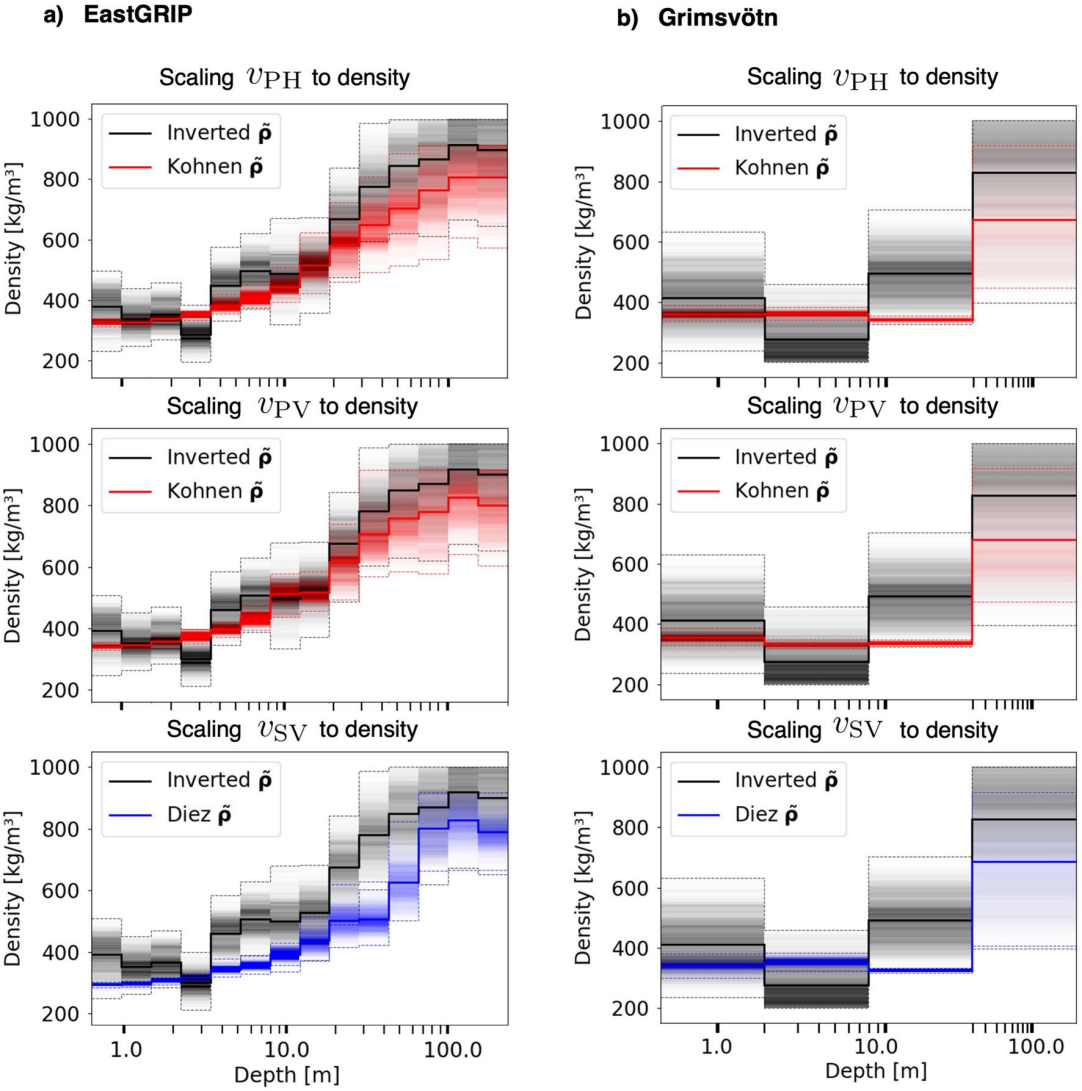
Comparison of depth-dependent probability distributions of density for the EastGRIP data set (**a**) and the Grímsvötn data set (**b**). Posterior mean densities, $\tilde{\rho }$, are shown as solid lines. Colored histograms represent the posterior wave speed samples scaled to density, using the relations of Kohnen^10^ for P-wave speed and the relation of Diez et al.^11^ for S-wave speed. Histograms in gray scale are the same as in Figs. 4 and 5, for density inferred directly from the surface wave dispersion data.

The posterior distributions of seismic wave speeds and density for the two experimental sites allow us to quantify the validity of two widely used scaling relations: Using data from various sites in Antarctica and Greenland, Kohne^10^ provides a relation between P-wave speed and density. A similar relation was derived for S-wave speed and density by Diez et al.^11^ on the basis of data from the Alpine glacier at Colle Gnifetti. Applying these scaling relations to the posterior wave speed samples, produces the posterior distributions for density, shown in the form of colored histograms in Fig. 6. These can be compared to the actual posterior distributions for density, constrained by the seismic data and displayed in gray scale in Fig. 6.

Since seismic wave speeds are mostly better constrained by seismic data than density, the uncertainty estimates for density distributions obtained from scaling relations are generally too optimistic. This is particularly pronounced for the Grímsvötn data inversion where P- and S-wave speeds in the firn layer have small posterior standard deviations in the range of tens of m/s. The general trend is that scaling relations under-predict the directly inferred density values by around 10 %, as order of magnitude. A notable exception are the shallow layers of comparatively low density, caused by human-made compaction near the surface. This feature is not captured by the scaling relations.

Most importantly, the differences between scaled and directly inferred density variations are significant. For both datasets and in several depth ranges, the two distributions shown in Fig. 6 have only small overlap, meaning that a density inferred by scaling falls outside the range of plausible density values inferred from the surface wave dispersion data. It follows that these data indeed carry valuable information about regional variations in glacier density that general-purpose scaling relations may not capture.

## Discussion

From a data perspective, the ability to constrain density directly rests on the availability of high-quality seismic recordings that contain higher-mode surface waves, and the framework is, in principle, transferable to other datasets if these higher modes are reliably identified. High-quality here refers to both high signal-to-noise ratio and dense spatial sampling. While the former was achieved by powerful human-made sources, the latter became possible thanks to the use of DAS. Ambient-noise studies, for example, often face challenges in capturing higher modes, particularly in cryospheric settings^20,23^, but higher modes have been extracted in other environments, such as submarine DAS experiments^53^. For datasets with narrower frequency content, weaker coupling, or reduced SNR, the resulting constraints on density and velocity structure may be weaker; nonetheless, the inversion framework itself remains applicable.

From a methodological perspective, the use of a fully nonlinear method, such as HMC, that permits the incorporation of honest and conservative prior knowledge, is essential. In regularized inversions, where prior knowledge is artificially strong to stabilise the method, information on model parameters with comparatively low sensitivity may easily be overwhelmed by subjective regularization choices^54–56^. The effect of nonlinearity is most apparent in the non-Gaussian shape of the posterior distributions, which may be skewed or multi-modal. A clear depth-dependent trend of nonlinearity, e.g., stronger or weaker deviations from Gaussian distributions with depth, is not apparent.

Notable limitations also have a data and a method component. The bandwidth of the available surface wave data, and in particular the lowest exploitable frequency, control the depth resolution. Below $\sim$100 m depth, sensitivity of all surface wave modes becomes too small for meaningful inferences on any model parameter. Although our method does not contain any explicit regularisation, the choice of the number and thickness of layers has an influence on the details of the results. As explained above, we selected the number of layers conservatively, i.e., as small as permitted by the requirement to approximately fit the data to within the observational errors. In this sense, our results are parsimonious and more plausible than alternative models with larger numbers of layers. The result that scaled densities are consistently lower than the directly inferred ones, seems to be particularly robust.

## Conclusions

The two key results of this work are the following: (i) Seismic surface wave data can directly constrain depth-dependent firn density. (ii) Commonly used scaling relations between seismic wave speeds and density tend to underestimate both density and its associated uncertainty. A recent study based on DAS data from the South Pole^12^, concluded that scaling relations overestimate firn density by 5–8%. Although the amplitude of the discrepancy is consistent with our results, the sign is not. This suggests that actual firn densities may vary on the order of ±10 %, which has consequences of similar magnitude for the derived mass contained in the firn layer.

## Data Availability

All data analyzed during the current study, as well as the collection of posterior HMC samples, are available on Zenodo under doi:10.5281/zenodo.15719335.
